# In Vitro Effects of Some Antibiotics on Purified β-Glucosidases from Rat Liver and Kidney Tissues

**DOI:** 10.3390/antibiotics14060563

**Published:** 2025-05-30

**Authors:** Hatibe Kara, Nihal Turkmen Alemdar

**Affiliations:** 1 Department of Biochemistry, Faculty of Veterinary Medicine, Bursa Uludag University, 16059 Bursa, Turkey; hatibekara@uludag.edu.tr; 2 Department of Biochemistry, Faculty of Veterinary Medicine, Balikesir University, 10100 Balikesir, Turkey; 3 Department of Medical Services and Techniques, Vocational School of Health Services, Recep Tayyip Edogan University, 53100 Rize, Turkey; 4 Department of Medical Biochemistry, Instute of Health Sciences, Balikesir University, 10100 Balikesir, Turkey

**Keywords:** antibiotics, β-glucosidase, inhibition, rat, liver, kidney

## Abstract

**Background:** Antibiotics are antimicrobial drugs used to treat and prevent infections. Unconscious use of antibiotics leads to many negative results. This study aimed to emphasize the negative aspects of antibiotics by determining their effects on purified enzymes. **Methods:** Beta glucosidase enzymes (BGLs) were purified from rat liver and kidney tissues using the sepharose-4B-LTyrosine-1-Naphthylamine hydrophobic interaction chromatography method. Liver BGL enzyme was purified 30.2-fold with a yield of 43.4%, while kidney BGL was purified 5.1-fold with a yield of 12.2%. Purified enzymes were visualized using SDS–PAGE. In vitro inhibition effects of ampicillin cefuroxime, amoxicillin–clavulanate, cefazolin sodium, gentamicin, and ceftriaxone antibiotics were determined on purified BGLs. **Results:** Ampicillin was found to inhibit rat liver and kidney BGLs competitively and uncompetitively, with IC50 values of 69.56 and 25.30 mM, respectively. Other antibiotics investigated did not significantly reduce liver BGL activity. Cefuroxime inhibited rat kidney BGL uncompetitively with IC50 values of 76.88 mM, while amoxicillin–clavulanate and cefazolin sodium inhibited it noncompetitively, with IC50 values of 41.32 and 98.81 mM, respectively. Gentamicin and ceftriaxone, whose effects were investigated, did not reduce kidney BGL activity. **Conclusions:** Some of the commonly used antibiotics reduce liver and kidney BGL activity, and this indicates that they may potentially impair metabolic functions. These results emphasize that caution should be exercised when using antibiotics.

## 1. Introduction

β-glucosidases (BGLs) hydrolyze the β-glycosidic bond between two glycone residues or glucose and an aryl/alkyl aglycone residue [1]. BGLs are present in almost all living forms and function in various processes, such as xenobiotic metabolism, intercellular signaling, and the storage of polysaccharides [2]. BGLs in humans [glucosylceramidase beta-1 (GBA1), glucosylceramidase beta-2 (GBA2), and glucosylceramidase beta-3 (GBA3)] are also called glucosylceramidases and play an effective role in important processes. GBA1 deficiency is associated with Gaucher disease, while GBA2 deficiency causes lactose intolerance. GBA3 is expressed in the mammalian liver, kidney, and small intestine and plays an important role in xenobiotic metabolism [3]. GBA3 human cytosolic β-glucosidase (EC. 3.2.1.21) is an enzyme belonging to the glycoside hydrolase family that exhibits broad substrate specificity and can hydrolyze substrates, such as β-D-glucoside, bound to a hydrophobic group [4]. It has a molecular weight of approximately 53 kDa and is found in mammals’ kidneys, liver, spleen, intestine, and lymphocytes. GBA3, which has significant activity against many common dietary compounds, including glycosides of flavonoids, cyanogens, phytoestrogens, and simple phenolics, has a special importance in xenobiotic metabolism [5]. Although no specific disease or metabolic association have been identified for cytosolic β-glucosidase, with ongoing studies, it has been shown that reduced or genetic silencing of GBA3 levels in some cancer types contributes to tumor proliferation by increasing tumor chemotherapeutic resistance [6,7,8,9].

Antibiotics are drugs used in treating and preventing bacterial infections through their mechanisms of action, namely the inhibition of pathogen cell wall synthesis, the disruption of cytosolic membrane permeability, and the inhibition of protein synthesis [10]. Antibiotic use rapidly spread after the first antibiotic compound was discovered in the early 1900s. The introduction of antibiotics into clinical use is considered one of the most important medical developments of the 20th century [11]. In addition to reducing morbidity and mortality caused by infectious diseases, antibiotics also make some problematic surgeries and procedures possible. However, the response to these agents may not always be as expected, which can lead to some adverse outcomes [12]. Genetic changes that occur due to the pharmacological properties of antibiotics also affect the genes encoding membrane transporters and enzymes involved in drug metabolism [13]. On the other hand, misusing these valuable compounds causes some infections to no longer be treated effectively, and antimicrobial resistance (AMR) rapidly increases. However, it is known that antibiotics that are metabolized in the liver and excreted through the kidneys cause severe metabolic damage in these tissues depending on the dose, plasma half-life, and duration of use [13,14,15]. DILI (drug-induced liver injury) refers to the damage caused by certain drugs in the liver. DILI can cause pathologies ranging from sudden liver enzyme increases to fatal complications such as severe hepatitis, liver failure or cirrhosis. Antibiotics are the most common group of drugs that cause DILI [16]. Although there is variability in the types of antibiotics associated with DILI, some commonly used antibiotics, such as amoxicillin–clavulanate and cefazolin, have been found to cause delayed liver damage. Studies also show that amoxicillin–clavulanate is the antibiotic that most commonly causes DILI [17].

The many different antibiotics that are widely available can be classified in various ways, for example, according to their microbial origin, mechanism of action, or chemical structure [18]. Ampicillin is a broad-spectrum penicillin derivative antibiotic containing a β-lactam ring which is generally used in treating respiratory tract, urinary tract, and gastrointestinal infections. Cefuroxime is a semi-synthetic cephalosporin antibiotic chemically similar to penicillin which exhibits broad-spectrum antibacterial activity due to its β-lactamase-resistant structure. Amoxicillin–clavulanate is a broad-spectrum antibiotic consisting of the aminopenicillin derivative amoxicillin and the β-lactamase inhibitor clavulanic acid and is preferred for respiratory tract infections and skin and soft tissue infections. Cefazolin is from the first-generation cephalosporin class and exhibits potent antibacterial activity against Gram-positive bacteria. Gentamicin is an antibiotic belonging to the aminoglycoside class. It has a bactericidal effect, especially against aerobic Gram-negative bacteria, by inhibiting protein synthesis, and is generally used in treating serious systemic infections and sepsis. Ceftriaxone is a third-generation cephalosporin, has a broad-spectrum antibacterial effect, and is widely used in the treatment of serious bacterial infections, such as meningitis, pneumonia, and sepsis [19,20].

In this study, the in vitro inhibitory effects of frequently prescribed antibiotics, such as ampicillin, cefuroxime, amoxicillin–clavulanate, cefazolin sodium, gentamicin, and ceftriaxone on BGLs purified from liver and kidney tissues were investigated. The inhibition types of antibiotics that have an inhibitory effect on enzyme activities were determined, and we aimed to draw attention to the harmful effects of the incorrect and frequent use of antibiotics.

The antibiotics used in the study were selected based on previous literature on frequency of use, known hepatotoxicity, and enzyme inhibition. Accordingly, the in vitro inhibitory effects of ampicillin, cefuroxime, amoxicillin–clavulanate, cefazolin, gentamicin, and ceftriaxone antibiotics, which clinicians commonly prescribe for the treatment of diseases, such as upper respiratory tract disorders, urinary tract infections, dental abscesses, *H. pylori* treatment, other gastrointestinal infections or infections that may develop after surgical interventions, sepsis, pneumonia, and meningitis, on BGLs purified from liver and kidney tissues were investigated [18,19]. This in vitro study aimed to draw attention to the possible side effects of inappropriate and frequent use of antibiotics and to guide new in vivo studies in terms of clinical applicability of this study. 

## 2. Results

### 2.1. Purification of BGLs from Rat Liver and Kidney Tissues by Hydrophobic Interaction Chromatography

**Figure 1:**
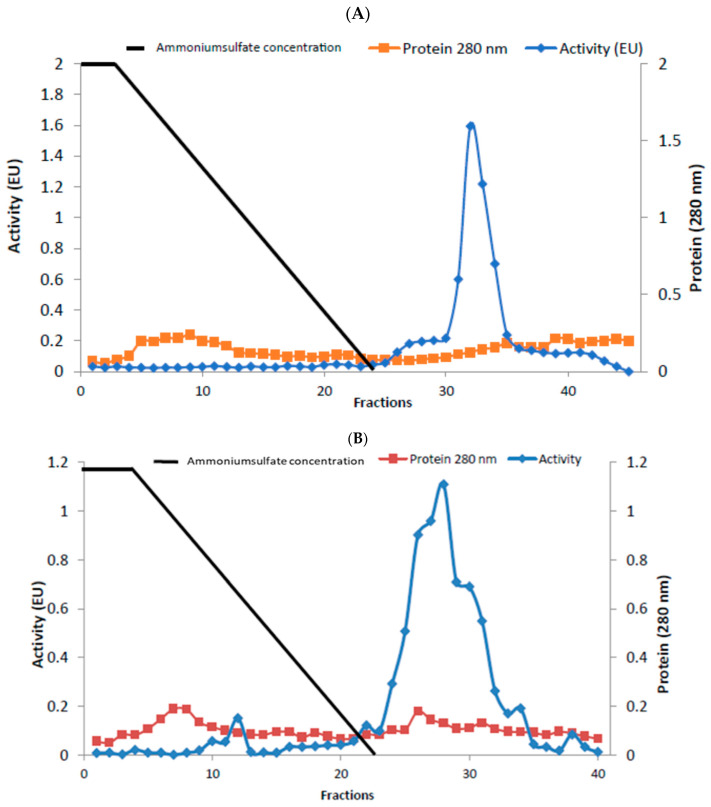
Purification of rat (**A**) liver (**B**) kidney β-glucosidases using a hydrophobic interaction chromatography column. Enzyme activity and total protein amount were determined for all samples. Enzyme activity was expressed in terms of *p*-nitrophenol (μm) released in the reaction mixture per minute under experimental conditions. The amount of protein was shown as the absorbance at 280 nm, and the straight line represents the ammonium sulfate salt gradient.

**Figure 2:**
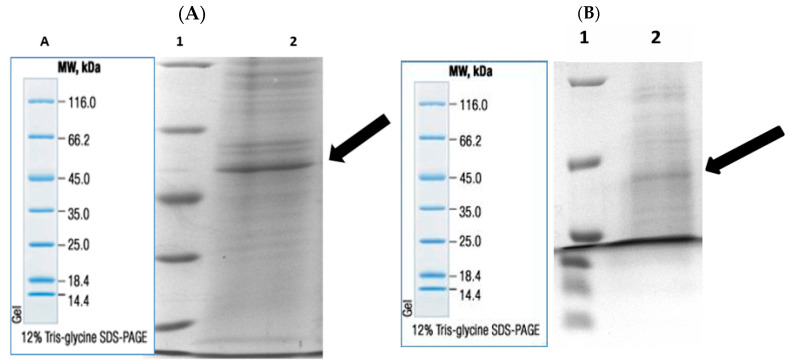
Post-purification rat (**A**) liver and (**B**) kidney BGLs by hydrophobic interaction chromatography. **Lane 1**: Molecular weight standards marker, (β-galactosidase (116.0 kDa), bovine serum albumin (66.2 kDa), egg albumin (45.0 kDa), lactate dehydrogenase (35.0 kDa), REase Bsp98I (*Escherichia coli*) (25.0 kDa), β-lactoglobulin (18.4 kDa), and lysozyme (14.4 kDa); **lane 2**: purified rat liver and kidney BGLs. A. Marker image.

Enzymes were purified by sepharose-4B-L-tyrosine-1-naphtylamine hydrophobic interaction chromatography. Rat liver and kidney tissue homogenates were precipitated in the 20–50% and 20–60% ammonium sulfate saturation range, respectively [21,22]. As a result of the purification process, liver BGL was purified 30.23-fold with a specific activity of 19.32 EU/mg protein and a yield of 43.48%. Kidney BGL was purified 5.08-fold with a specific activity of 10.94 EU/mg protein and a yield of 12.21% (Table 1). When the purification graphs were examined, it was observed that the rat liver BGL was eluted in fractions 31–34 (Figure 1A), while kidney BGL was eluted from the column in fractions 24–34 (Figure 1B). Eluted enzymes were visualized on SDS–PAGE to evaluate their purity, and protein bands were visualized (Figure 2A,B, Supplementary Materials, Figure S1). As a result of the analyses, the approximate molecular weights of liver and kidney BGL enzymes were determined to be 58 kDa.

### 2.2. In Vitro Effects of Antibiotics on Rat Liver and Kidney BGLs

**Figure 3:**
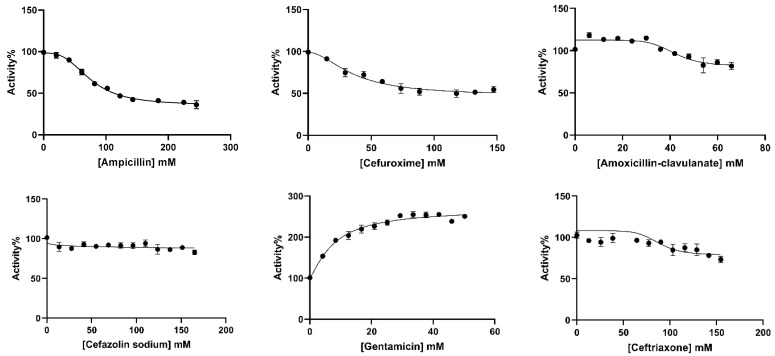
IC_50_ plots of antibiotics investigated for in vitro effects on rat liver BGL. The plots were plotted with GraphPad Prisim software.

**Figure 4:**
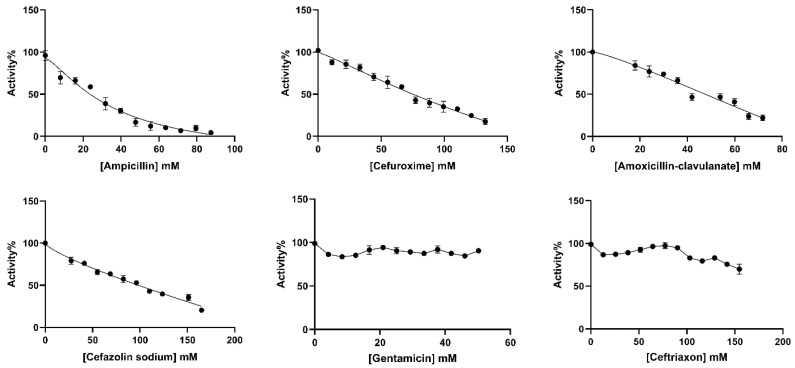
IC_50_ plots of antibiotics investigated for in vitro effects on rat kidney BGL. The plots were plotted with GraphPad Prisim software.

**Figure 5:**
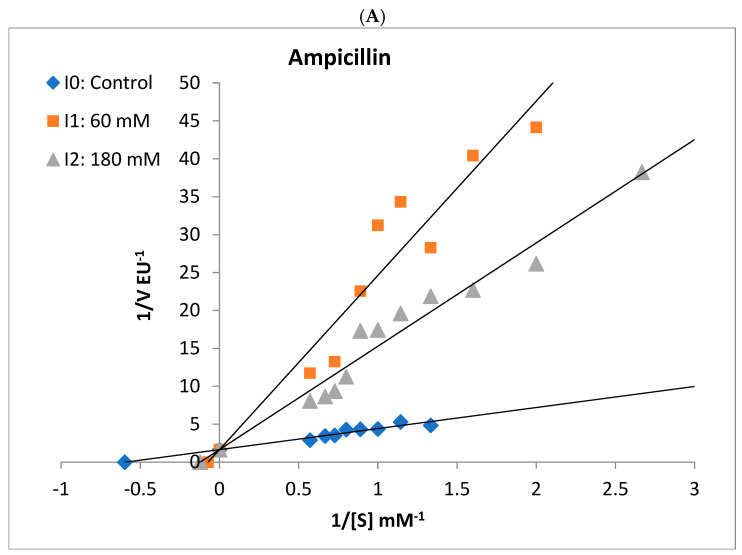

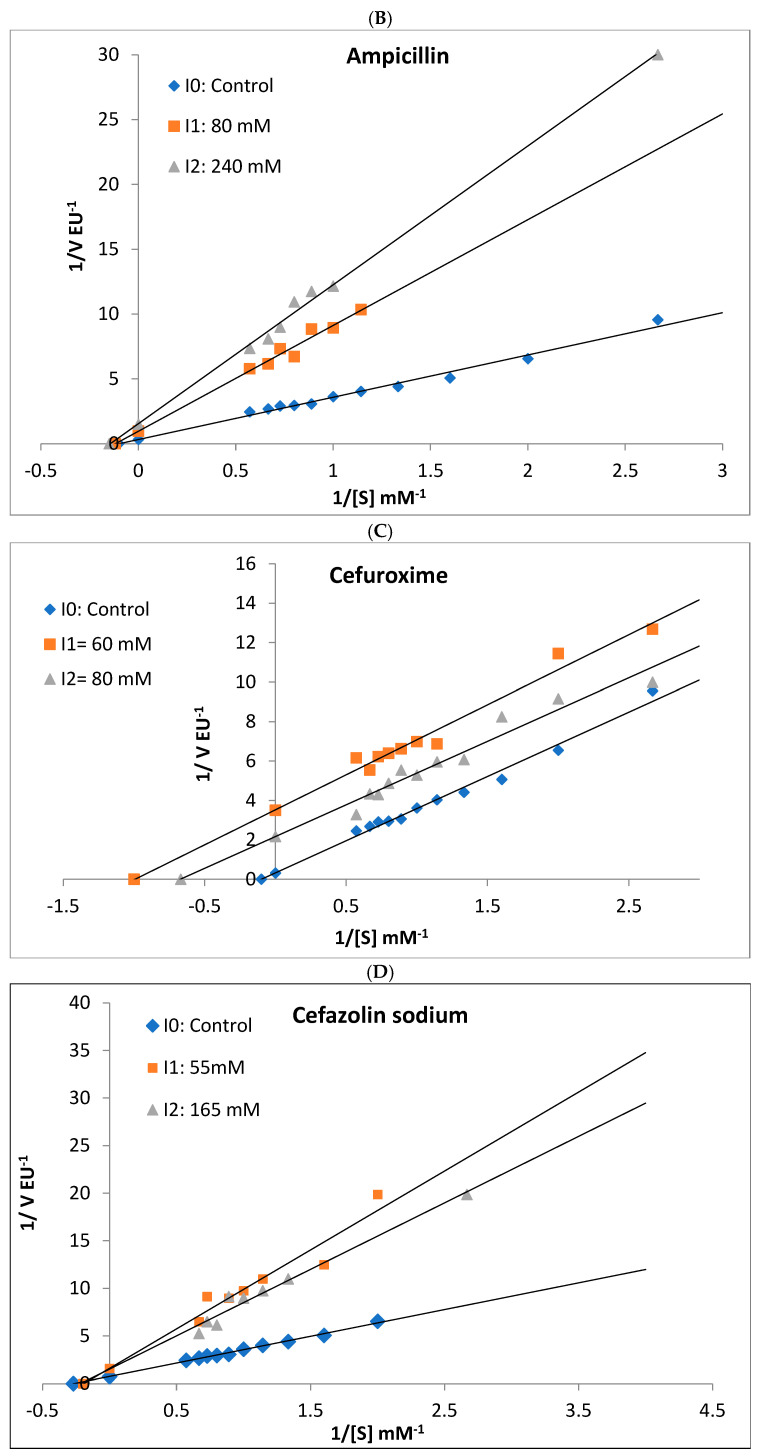

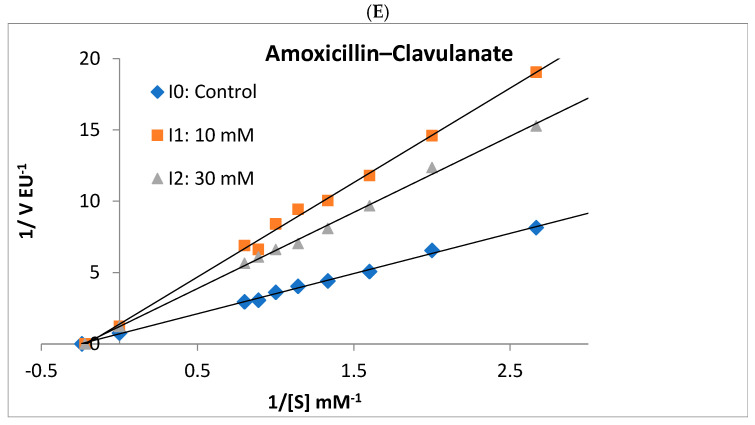
Lineweaver–Burk plots used to determine the effect of different antibiotics on the activities of rat liver and rat kidney BGLs. Ampicillin competitively inhibited liver BGL (**A**) and noncompetitively inhibited kidney BGL (**B**). Kidney BGL enzyme was inhibited by cefuroxime (**C**) in the uncompetitive type, while cefazolin sodium (**D**) and amoxicillin–clavulanate (**E**) in the non-competitive type.

The in vitro effects of ampicillin, cefuroxime, amoxicillin–clavulanate, cefazolin sodium, gentamicin, and ceftriaxone antibiotics on BGLs purified from rat liver and kidney tissues were analyzed. IC_50_ values, defined as the antibiotic concentration that reduced enzyme activity by 50% (Figure 3 and Figure 4), were calculated using GraphPad Prism software (9.5.1. version) (Table 2). The types of inhibition of antibiotics that inhibit rat liver and kidney BGLs were determined based on Lineweaver–Burk kinetic analysis, and *K_i_* values were calculated from the graphs (Figure 5).

It was determined that gentamicin, an aminoglycoside antibiotic, and cefuroxime, cefazolin sodium, amoxicillin–clavulanate, and ceftriaxone antibiotics belonging to the cephalosporin class did not show a significant inhibitory effect on rat liver BGL activity. However, increased enzyme activity was observed in the presence of gentamicin. The IC_50_ value of ampicillin, a penicillin-class antibiotic, was calculated as 69.56 mM (Figure 3, Table 2). It was determined that ampicillin showed a competitive inhibition mechanism on rat liver BGL, and the inhibitory binding constant *K_i_* was calculated as 14.30 ± 3.35 mM (Figure 5A).

Ampicillin, cefuroxime, amoxicillin–clavulanate, and cefazolin sodium, were determined to inhibit the enzyme, and the IC_50_ values were calculated as 25.30, 76.88, 41.32, and 98.81 mM, respectively (Table 2). It was determined that ampicillin exhibited a noncompetitive inhibition mechanism on rat kidney BGL, and the *K_i_* value was calculated as 28.37 ± 6.35 Mm (Figure 5B). Amoxicillin–clavulanate and cefazolin sodium showed noncompetitive type of inhibition and had *K_i_* values of 27.19 ± 12.04 mM and 101.90 ± 47.09 mM, respectively (Figure 5). Cefuroxime was found to have the uncompetitive type of inhibition, and its *K_i_* value was determined as 18.65 ± 12.65 mM. It was observed that gentamicin and ceftriaxone antibiotics did not significantly inhibit rat kidney BGL activity, similar to rat liver BGL (Figure 4).

## 3. Discussion

Although antibiotics have been among the most commonly used therapeutic drugs in the world since their discovery, the serious side effects they create and the antimicrobial resistance crisis that has developed due to their misuse limit their use worldwide [23]. Antibiotics disrupt the activity of the beneficial microbiome in the intestinal microbiota and can lead to side effects, such as hepatotoxicity and nephrotoxicity. Current studies reveal that antibiotic use can cause serious damage to the liver and kidneys, especially when administered for a long time or at high doses [24]. Since the liver is one of the main organs where antibiotics are metabolized, some antibiotics can cause oxidative stress and cellular dysfunction in hepatocytes [25]. Prospective and retrospective studies, especially on DILI, show that antibiotics are the most common type of drug that causes idiosyncratic liver damage [17]. In addition, studies on patients with drug-induced acute liver failure (ALF) have shown that antibiotics are the most common cause of drug-induced liver failure or are among the most common drug classes that cause ALF [26,27]. Another organ affected by the side effects of antibiotics is the kidneys. Since the kidneys are responsible for the filtration and excretion of antibiotics, the use of antibiotics can lead to acute renal failure and chronic kidney damage in the long term due to their toxic effects on the renal tubules. The resulting damage may lead to severe renal failure, especially in sensitive patient groups [28,29]. GBA3 is a cytosolic β-glucosidase effective in xenobiotic metabolism, primarily localized in the kidney, liver, spleen, intestine, and lymphocytes of mammals [30]. Cytosolic BGL has significant activity against many common dietary xenobiotics in humans, including phytoestrogen glycosides, flavonoids, simple phenolics, and cyanogens [5]. In recent years, important results have revealed that GBA3 is associated with different metabolic diseases. It is suggested that GBA3 supports fatty acid oxidation and alleviates non-alcoholic fatty liver disease. Research shows that inflammatory factors are at lower levels in rats receiving GBA3 supplementation [31].

Studies investigating the effects of drugs can be designed as in vivo or in vitro experiments. As is known, in vitro studies have many limitations compared to in vivo studies. In vitro studies are initiated independently of the physiological, metabolic, endocrinological, and neurological processes of the living organism in order to avoid the possible negative effects of an in vivo study. [32]. The present study focuses on the in vitro effects of commonly used antibiotics on liver and kidney BGLs. For this purpose, BGLs were purified from rat liver and kidney tissues. Chromatographic methods are frequently employed for enzyme purification [33,34,35,36]. The use of chromatographic techniques for BGL purification from various mammalian tissues has been well documented [37,38]. However, in this study, rat liver and kidney BGLs were purified for the first time using sepharose-4B-L-tyrosine-1-naphthylamine hydrophobic interaction chromatography.

This specific hydrophobic gel has been previously used for the purification of human paraoxonase 1 (PON1) [21,39] as well as for the purification of PON1 from bull sperm [40]. Additionally, the same hydrophobic gel has been successfully utilized to purify BGLs from plant sources, including olive [22], broccoli [41], and mandarin [42], as well as from animal sources, such as insects [43] and lamb liver [44]. These findings suggest that the gel may also be effective in purifying BGLs from rat tissues. Following the purification process, rat liver BGL was purified 30.2-fold with a specific activity of 19.32 EU/mg and a yield of 43.4%. In contrast, kidney BGL exhibited a lower specific activity of 10.94 EU/mg, achieving 5.1-fold purification with a 12.5% yield. These results indicate that the applied method was more effective in purifying liver BGL than kidney BGL.

A comparison with previous studies reveals the use of various chromatographic techniques for the purification of mammalian BGLs. Lambert et al. [45] purified BGL from porcine liver using multiple chromatographic steps, obtaining a specific activity of 15.8 U/mg, with a 12.2% yield and 1436-fold purification, through Superdex 200 HR column chromatography. In another study, human liver cytosolic β-glucosidase was purified using a combination of cation exchange chromatography and octyl-sepharose hydrophobic chromatography [5]. Furthermore, Matern and colleagues successfully purified bile acid BGL (GBA2) from human liver microsomes via DEAE–Trisacryl chromatography, Mono Q chromatography, and affinity chromatography [37]. Similarly, porcine liver BGL was purified using affinity chromatography, achieving a 24.5% yield, 161-fold purification, and a specific activity of 6280 U/mg [38]. Studies on kidney BGL purification are more limited. Pierce et al. [46] purified BGL from primate kidney using ion-exchange chromatography, whereas Pocsi et al. [47] purified BGL from porcine kidney via gel filtration chromatography. Additionally, Paez de Cadene et al. [48] purified BGL from rabbit kidney tissue through a combination of affinity chromatography, DEAE cellulose chromatography, Sephadex G-200 gel chromatography, and hydroxyapatite chromatography, obtaining a 126-fold purification with a 21% yield. In our study, the hydrophobic gel sepharose-4B-L-tyrosine-1-naphthylamine provided a higher yield (43.4%) for rat liver BGL compared to purification yields reported for porcine and human liver BGLs [38,45]. Moreover, our findings indicate that this method was more efficient in purifying liver BGL than kidney BGL. These results suggest that the hydrophobic interaction chromatography technique utilizing the laboratory-synthesized sepharose-4B-L-tyrosine-1-naphthylamine column may be effectively employed for BGL purification from mammalian tissues.

The rat liver and kidney BGLs, purified in two steps, were analyzed by SDS–polyacrylamide gel electrophoresis (SDS–PAGE) to determine their molecular weights. Bands were observed at approximately 58 kDa (Figure 2A,B). Among the three different β-glucosidase isoforms found in mammals, the molecular weight of cytosolic β-glucosidase (GBA3) varies depending on the tissue and organism, with reported values ranging between 53 and 59 kDa [5,45,47,49]. However, previous studies have indicated that BGLs may exhibit abnormal migration patterns on polyacrylamide or agarose gels, leading to variations in their apparent molecular weight [38,50]. The presence of additional bands at different kDa positions may be attributed to the purification method and degree of purification, and it is expected that optimizing these parameters could eliminate these discrepancies. Given that the purified rat liver and kidney enzymes exhibited a molecular weight of 58 kDa on SDS–PAGE, they were identified as GBA3 isoforms, confirming their classification as cytosolic BGLs.

The in vitro effects of commonly prescribed antibiotics—ampicillin, cefuroxime, amoxicillin–clavulanate, cefazolin sodium, gentamicin, and ceftriaxone—on the activities of purified rat liver and kidney BGLs were investigated. Ampicillin was found to competitively inhibit rat liver BGL, with an IC_50_ value of 62.97 mM (Table 2, Figure 5). Competitive inhibition suggests that ampicillin competes with the substrate for the active site, binding to the enzyme and reducing its catalytic activity. As a result, increasing ampicillin concentrations in the environment are expected to decrease the catalytic efficiency of the enzyme. Given that cytosolic BGL plays a critical role in xenobiotic metabolism in the liver, these findings suggest that ampicillin may negatively impact this enzymatic process. Ampicillin is an antibiotic generally known for not inhibiting liver and kidney enzymes and not causing damage. However, in this study, it was shown that it inhibited both tissue BGLs. However, it should be taken into consideration that the ampicillin concentration used here was very high. The presence of 40 mM ampicillin in the medium caused a 10% loss in liver BGL activity. This study reveals the effects of a high dose of ampicillin.

Among the other antibiotics tested, none inhibited BGL activity by more than 80%, and, interestingly, gentamicin increased enzyme activity within the tested concentration range. A study conducted by Güller et al. [29] demonstrated that cefazolin sodium did not inhibit glutathione reductase (GR) activity purified from mouse liver, whereas amoxicillin and gentamicin exhibited competitive and noncompetitive inhibition, respectively. The effect of cefazolin sodium observed in the present study aligns with these findings. Gentamicin belongs to the aminoglycoside antibiotic class, characterized by highly polar structures containing amino sugars [51]. Despite being associated with ototoxicity and nephrotoxicity, our study demonstrated that gentamicin enhanced the activity of BGL purified from rat liver. Similarly, gentamicin has been reported to increase the activity of glutathione reductase (GR) purified from human erythrocytes [52]. These findings do not contradict the existing literature but highlight the potential modulatory effects of aminoglycosides on enzymatic activity.

The rat kidney BGL was inhibited by ampicillin, cefuroxime, amoxicillin–clavulanate, and cefazolin sodium, with IC_50_ values of 25.30 mM, 76.88 mM, 41.32 mM, and 98.81 mM, respectively (Table 2). In contrast, gentamicin and ceftriaxone did not exhibit inhibitory effects on the enzyme. Ampicillin, amoxicillin–clavulanate, and cefazolin sodium inhibited rat kidney BGL via noncompetitive inhibition, whereas cefuroxime acted as an uncompetitive inhibitor (Figure 5). Noncompetitive inhibition indicates that ampicillin, amoxicillin–clavulanate, and cefazolin sodium can bind to both the free enzyme and the enzyme–substrate complex without directly competing with the substrate. In such cases, increasing substrate concentration cannot overcome inhibition, meaning that as long as these antibiotics are present, BGL activity will remain suppressed. On the other hand, the uncompetitive inhibition observed with cefuroxime suggests that the inhibitor does not prevent substrate binding but instead binds to the enzyme–substrate complex, reducing catalytic efficiency. This type of inhibition is particularly significant in intracellular metabolic regulation, as high substrate concentrations do not mitigate the inhibitory effect. From a pharmacological perspective, uncompetitive inhibitors have the potential to permanently reduce enzyme activity, making them relevant in drug development strategies. In line with previous research, gentamicin did not inhibit rat kidney BGL, a finding consistent with results from Güller et al. [29], who reported that gentamicin and cefazolin sodium did not inhibit glutathione reductase (GR) activity purified from mouse kidney tissue, whereas amoxicillin inhibited GR via noncompetitive inhibition with an IC_50_ of 86 mM. In our study, amoxicillin–clavulanate exhibited stronger inhibition, with an IC_50_ of 41.32 mM, suggesting that it is more potent than amoxicillin alone. Notably, amoxicillin–clavulanate has been reported to be more toxic than amoxicillin [53]. A study by Çiftçi et al. [54] demonstrated that sodium ampicillin, sodium cefuroxime, sodium cefazolin, and gentamicin sulfate inhibited glucose-6-phosphate dehydrogenase (G6PD) purified from human erythrocytes, with IC_50_ values of 105 mM, 6.7 mM, 125 mM, and 12 mM, respectively. Compared to our findings, ampicillin inhibited rat liver BGL at 70 mM and rat kidney BGL at 25 mM, indicating that its inhibitory effect on human erythrocyte G6PD was weaker than on rat kidney BGL. Additionally, cefuroxime sodium exhibited stronger inhibition on human erythrocyte G6PD than on rat kidney BGL. Beydemir et al. [55] reported that gentamicin sulfate noncompetitively inhibited G6PD purified from sheep erythrocytes. In contrast, our study showed that gentamicin had no inhibitory effect on rat liver or kidney BGLs, despite its reported inhibitory effects on human and sheep erythrocyte G6PD. Cefazolin sodium inhibited both human erythrocyte G6PD and rat kidney BGL via noncompetitive inhibition, suggesting a similar mechanism of action.

Studies investigating the effects of macrolide antibiotics on glucosidases have shown that erythromycin and clarithromycin exhibit stronger inhibitory effects than azithromycin [56]. Furthermore, Adem et al. [57] reported that cefuroxime noncompetitively inhibited glucose-6-phosphate dehydrogenase (G6PD), 6-phosphogluconate dehydrogenase (6PGD), and glutathione reductase (GR) purified from rat lung tissue, while gentamicin competitively inhibited these enzymes. In our study, cefuroxime exhibited uncompetitive inhibition of rat kidney BGL, whereas gentamicin had no effect on enzyme activity. Although gentamicin is widely known for its nephrotoxicity, it did not alter rat kidney BGL activity in our study. This finding highlights the specificity of enzyme–antibiotic interactions and suggests that not all nephrotoxic antibiotics directly affect renal BGL activity.

Compounds ingested orally from plants, which may be metabolized into potentially toxic or beneficial metabolites, undergo biotransformation through xenobiotic metabolism in the body. In this process, β-glucosidases are particularly hydrolyzed, and cytosolic β-glucosidase (GBA3) plays a critical role in this mechanism [5]. The association between GBA3 and various metabolic disorders has been extensively investigated in recent years, yielding significant findings. Studies suggest GBA3 supports fatty acid oxidation and alleviates non-alcoholic fatty liver disease. Additionally, in rats supplemented with GBA3, reduced levels of inflammatory factors were observed, and overexpression of GBA3 was reported to decrease reactive oxygen species (ROS) production and cellular apoptosis rates [31].

Research on the physiological roles of mammalian BGLs has recently increased. In this context, the present study is a pioneering report that evaluates the in vitro effects of commonly used antibiotics on purified rat liver and kidney BGLs. However, living organisms are highly complex biological systems, and the results obtained from in vitro studies may not always directly translate to in vivo conditions. Therefore, validating these findings in in vivo models will facilitate a more comprehensive understanding and yield clinically relevant insights.

The study had some limitations. In the literature, SDS–PAGE images and enzyme purification coefficients in different studies conducted with similar enzyme purification methods seem to be more successful compared to this study [5,37,38]. In this study, the purification process was only performed by hydrophobic gel chromatography, and if it was possible to add one more step (ionic exchange, gel filtration chromatography, etc.) to the purification process, a cleaner purification could have been obtained.

## 4. Materials and Methods

### 4.1. Chemicals

*Para-nitrophenyl glucopyranoside* (*p*-NPG) (Cat. No. N7006, Sigma Aldrich, St. Louis, MO, USA), N, inorganic salts, protein assay reagents, Triton X-100 (Cat. No. T8787, Taufkirchen, Germany), sucrose (Cat. No. S8501, Sigma-Aldrich, Taufkirchen, Germany), and the chemicals for electrophoresis used in experimental studies were supplied by Sigma Aldrich. The antibiotics whose effects were investigated were obtained from local pharmacies. The chromatography gel, sepharose-4B-L-tyrosine-1-naphthylamine, was synthesized in the Biochemistry Laboratory of Balıkesir University, Faculty of Veterinary Medicine, according to the method of Sinan et al. [21].

### 4.2. Animals

The approval for this study was obtained from the Local Animal Care Committee of Çanakkale Onsekiz Mart University (Decision number: 2015/08-24). Five male Wistar-Albino rats (15–17 weeks old, 180–220 g in body weight) were used for this study. The animals were raised under standard feed and living conditions. Animal experiments were carried out in Çanakkale Onsekiz Mart University Experimental Research Application and Research Center (COMUDAM/Türkiye). After standard experimental animal laboratory conditions and a 12 h fasting period, the rats were anaesthetized intraperitoneally with 50 mg/kg ketamine and 10 mg/kg xylazine. The tissue samples were placed in Eppendorf tubes, frozen rapidly, and stored at −80 °C until measurements were performed.

### 4.3. Enzyme Extraction

Crude extractions to obtain BGL enzymes from rat liver and kidney tissues were carried out according to the method of Mattern et al. [37]. Crude extractions were performed separately from rat liver and kidney tissue samples. The tissue was suspended at four times its weight in 5 mM Tris–HCl buffer pH 7.4, containing 0.25 M sucrose. The suspension was homogenized with a homogenizer (Stuart SHM-1, Fort Wayne, India) at 10.500 min^−1^ revolutions for 6 min and then centrifuged at 1000 rpm for 10 min to remove the precipitated large particles and nuclei. The supernatant was centrifuged at 16,000 rpm for 60 min. Then, the pellet was suspended in 5 mM Tris–HCl pH, 7.4, containing 0.25 M sucrose. Then, 0.75% Triton X-100 was added to this suspension and mixed by vortexing (Stuart SA8, Fort Wayne, India) to extract BGLs from the suspended pellet. The suspension was kept at a temperature of +4 °C for 30 min and then centrifuged at 16,000 rpm for 60 min. The supernatant was used as a crude extract.

### 4.4. Purification of BGLs from the Rat Liver and Kidney by Hydrophobic Interaction Chromatography with Sepharose 4B-L-Tirozin 1-Naphtylamine

Purification was accomplished by hydrophobic interaction chromatography using a sepharose-4B-L-tyrosine-1-naphthylamine column with minor modifications according to the method applied by Kara and colleagues [22]. Before purification, a hydrophobic column (1.0 cm × 7.0 cm) was equilibrated with 50 mM sodium phosphate buffer (pH 6.8) containing 1 M ammonium sulfate.

Crude extracts were precipitated with solid ammonium sulfate at 20–50% and 20–60% saturation ranges for partial purification of liver and kidney BGL enzymes, respectively. Ammonium sulfate saturation degrees were optimized before the experiments [21,22]. After the centrifugation of both kidney and liver tissues at 15,000 rpm for 30 min, the resulting pellets were dissolved in a minimum volume of 50 mM (pH 6.8) sodium phosphate buffer. The enzyme solution obtained was eluted with the same buffer at a flow rate of 50 mL/h and a 1.0–0.0 M (NH_4_)_2_SO_4_ gradient. Fractions were collected in 2 mL volumes in microcentrifuge tubes and elution was continued until an absorbance value of 0.02 was reached at a 280 nm wavelength. Each fraction was analyzed for BGL activity with the substrate *p*-NPG and qualitative protein determination was carried out. Fractions showing the highest BGL activity were combined and used as purified enzymes in the in vitro antibiotic studies.

### 4.5. SDS–PAGE Analysis

Sodium dodecyl sulfate–polyacrylamide gel electrophoresis (SDS–PAGE) was performed according to the procedure described by Laemmli [58] to confirm the homogeneity of the purified BGLs. Purified protein samples were fractionated in Tris–glycine–SDS buffer at 150 volts on 10% SDS–PAGE gels using the Minigel system (Bio-Rad Laboratories, Thane, India). Gels were then stained with Coomassie Brillant Blue R-250 (Sigma, Kawasaki, Japan) and treated with decolorizing solution (7.5% (*v*) acetic acid, 5% (*v*) methanol in water) to detect protein bands. Protein bands were visualized with a gel imaging system (UVP, Analytik Jena Company, Jena, Germany). Thermo Fisher protein marker (Cat. No. 26610, Waltham, MA, USA) was used to estimate the size of the protein bands and, the protein weights were estimated by plotting LogMW against Rf measurements calculated from the gel (Thermo Fisher Scientific, Waltham, MA, USA).

### 4.6. Enzyme Assay and Protein Determination

Rat liver and kidney BGL activities were determined spectrophotometrically using a ThermoFisher Scientific Multiskan™ FC microplate (Thermo Fisher Scientific, Waltham, MA, USA). *p*-NPG dissolved in 50 mM of pH 5.5 Na–Ac buffer was used as the substrate. In this method, enzyme activity was determined by photometrically measuring the determination of *p*-nitrophenol (*p*-NP) released in the presence of BGLs spectrophotometrically at 405 nm. Calculations were made with the standard curve of *p*-NP (Cat. No. 241326, Sigma) prepared at 405 nm. One enzyme unit (EU) was defined as the amount of μmol *p*-nitrophenol released per minute in the reaction medium under the specified experimental conditions [22].

Quantitative protein amount was performed using bovine serum albumin (Cat. No. A8806, Sigma) as a standard protein according to the Lowry method [59]. Qualitative protein determination was carried out by absorbance determination at 280 nm.

### 4.7. Determination of In Vitro Effects of Antibiotics

The half-maximal inhibitory concentration (IC50) of antibiotics was determined using a constant concentration of *p*-NPG of 1.75 mM. At least ten different concentration ranges of each antibiotic were tested to evaluate the in vitro effects of ampicillin (5–250 mM), amoxicillin–clavulanate (5–70 mM), cefuroxime (5–150 mM), cefazolin sodium (10–165 mM), ceftriaxone (10–160 mM), and gentamicin (5–50 mM). Enzyme activity was measured in the presence of different antibiotic concentrations, and BGL activity in the medium without antibiotics, which was accepted as the control group, was determined as 100% reference value. Enzyme activity results were expressed as % activity and the inhibitory concentration (IC_50_) that reduced enzyme activity by 50% was calculated using GraphPad Prism software. All experiments were performed in three replicates for each antibiotic concentration.

Lineweaver–Burk double-reciprocal plots were used to determine the inhibitory effect of antibiotics and the inhibition mechanisms [60]. The curve obtained when there was no inhibitor in the reaction medium was accepted as the control (I_0_); the intersection points of the control curve with the lines drawn in the presence of the inhibitor at two different concentrations were analyzed. In line with these intersection points, the relevant inhibition types were determined, and the inhibitory constants (*K_i_*) of the inhibitors were calculated.

## 5. Conclusions

Antibiotics are a class of compounds that can cause liver and kidney damage, and their effects on enzymatic processes may influence the function of these organs. In this study, the inhibitory effects of commonly prescribed antibiotics, including ampicillin, amoxicillin–clavulanate, cefuroxime, cefazolin sodium, gentamicin, and ceftriaxone, on BGLs purified from rat liver and kidney tissues were evaluated. Our results indicate that ampicillin significantly reduced BGL activity in both liver and kidney tissues, while cefuroxime, amoxicillin–clavulanate, and cefazolin sodium inhibited only kidney BGL activity. These findings suggest that certain antibiotics may negatively impact xenobiotic metabolism. Furthermore, the inhibitory effects of specific antibiotics on BGLs raise concerns about their potential toxic effects on metabolic processes.

The findings of this study highlight the need for a more detailed investigation of the effects of antibiotics on enzyme activity, which could contribute to dose determination and clinical applications. Additionally, this study draws attention to the potential harm associated with the misuse of antibiotics, emphasizing the importance of rational antibiotic use in clinical practice.

## Figures and Tables

**Table 1:** Purification table of rat liver and kidney β-glucosidases.

Tissue	Steps	Activity (EU/mL)	Total Activity (EU)	Protein (mg/mL)	Total Protein (mg)	Specific Activity (EU/mg)	Yield (%)	Purification Factor (Fold)
Liver	Crude extract	1.65	9.95	2.59	15.56	0.63	100.00	1.0
	AS	1.97	6.90	0.53	0.53	3.71	69.39	5.81
	HIC	2.16	4.32	0.11	0.22	19.32	43.43	30.23
Kidney	Crude extract	4.09	24.56	1.89	11.39	2.15	100.00	1.0
	AS	6.23	19.32	1.90	5.91	3.26	78.68	1.52
	HIC	1.50	3.01	0.13	0.27	10.94	12.21	5.08

AS: Ammonium sulfate precipitation; HIC: hydrophobic interaction chromatography.

**Table 2:** Effects of the investigated antibiotics on liver and kidney β-glucosidase activity.

Enzymes	Antibiotics	IC_50_ (mM)	*K_i_* (mM)	Inhibition Type
Liver BGL	Ampicillin	69.56	14.30 ± 3.35	Competitive
	Cefuroxime	N/A	N/A	NI
	Amoxicillin–clavulanate	N/A	N/A	NI
	Cefazolin sodium	N/A	N/A	NI
	Gentamicin	N/A	N/A	NI
	Ceftriaxone	N/A	N/A	NI
Kidney BGL	Ampicillin	25.30	28.37 ± 6.35	Noncompetitive
	Cefuroxime	76.88	18.65 ± 12.65	Uncompetitive
	Amoxicillin–clavulanate	41.32	27.19 ± 12.04	Noncompetitive
	Cefazolin sodium	98.81	101.90 ± 47.09	Noncompetitive
	Gentamicin	N/A	N/A	NI
	Ceftriaxone	N/A	N/A	NI

N/A: Indicates not applicable, NI: No observed inhibition.

## Data Availability

The datasets presented in the current study are available from the corresponding author upon reasonable request.

## Supplementary Materials

The following supporting information can be downloaded at: https://www.mdpi.com/article/10.3390/antibiotics14060563/s1, Figure S1. Purified rat (A–C) liver and (D) kidney BGLs at the end of the hydrophobic interaction chromatography on SDS–PAGE gel images (original photographs).

## Abbreviations

BGL	β-glucosidase
GR	glutathione reductase
G6PD	glucose-6-phosphate dehydrogenase
*p*-NP	*para*-nitrophenol
*p*-NPG	*para*-nitrophenyl-β-D-glucopyranoside
