# Supplementary material for: In Vitro Effects of Some Antibiotics on Purified β-Glucosidases from Rat Liver and Kidney Tissues
Source: Antibiotics (Basel). 2025 May 30;14(6):563. doi: 10.3390/antibiotics14060563 (PMC12189931; doi:10.3390/antibiotics14060563)
Supplement: Supplementary file 1 [file antibiotics-14-00563-s001.zip › antibiotics-3581625-supplementary.pdf]

(A)

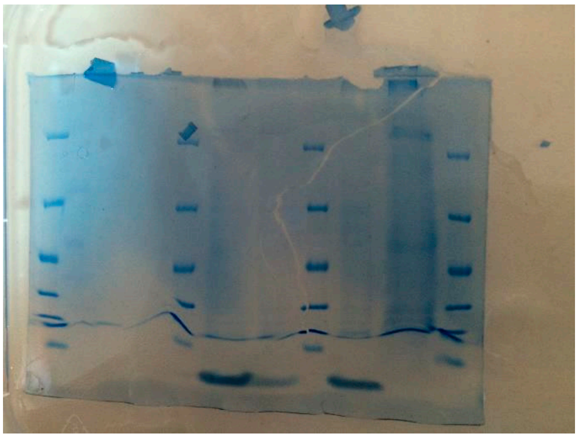

(B)

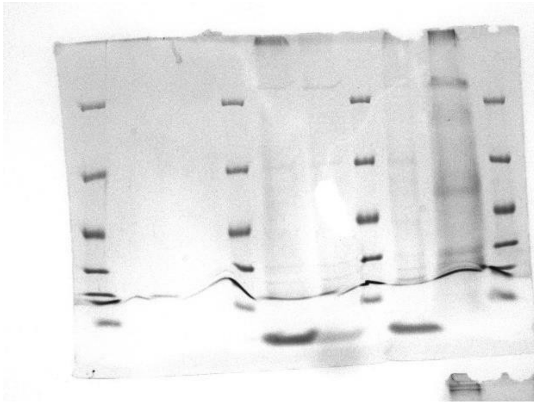

(C)

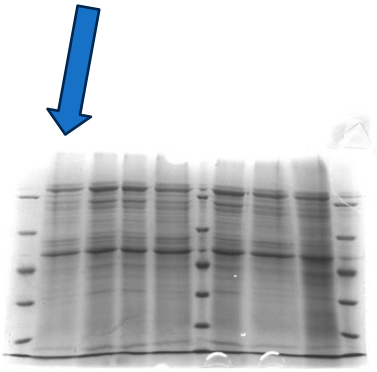

(D)

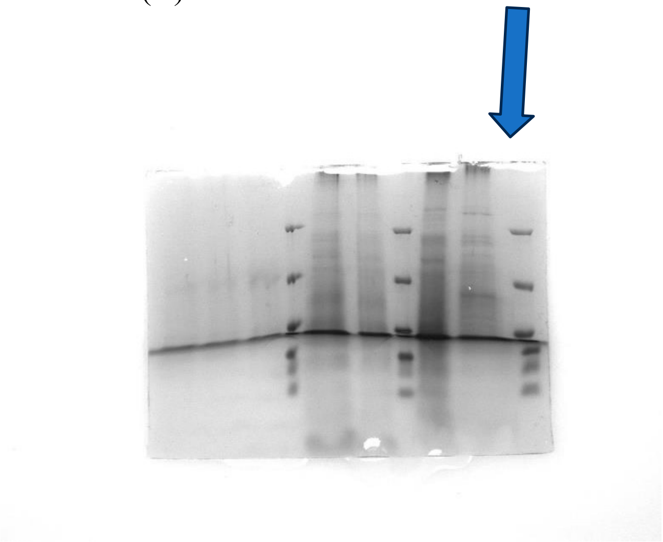

Figure S1. Purified rat (A, B, and C) liver and (D) kidney BGLs at the end of the hydrophobic interaction chromatography on SDS–PAGE gel images (original photographs).

Images A, B, and C are the of rat liver purified BGL enzyme, but although images A and B are identical, image C was obtained from another set (A, original raw figure; B, raw figure). D is an SDS-PAGE gel image of the rat kidney purified BGL enzyme (raw figure).

The images shown with arrows are the images used in the manuscript.
